# New paths in post-graduate medical training in general practice – 8 years of experience with the pilot project Verbundweiterbildung^plus^ Baden-Württemberg

**DOI:** 10.3205/zma001139

**Published:** 2017-11-15

**Authors:** Simon Schwill, Julia Magez, Stefanie Joos, Jost Steinhäuser, Thomas Ledig, Aline Rubik, Wilhelm Niebling, Joachim Szecsenyi, Elisabeth Flum

**Affiliations:** 1 University Hospital Heidelberg, Department of General Practice and Health Services Research, Heidelberg, Germany; 2 University Hospital Tübingen, Institute of General Practice and Interprofessional Care, Tübingen, Germany; 3 University Hospital Schleswig-Holstein, Campus Lübeck, Institute of Family Medicine, Lübeck, Germany; 4 University Hospital Freiburg, Department of General Practice, Freiburg, Germany

**Keywords:** General medicine, specialized medical training, mentoring, train-the-trainer, networked training programs, accompanying seminars

## Abstract

**Background: **In face of the looming shortage of general practitioners, primary healthcare providers and post-graduate training in general practice are increasingly becoming part of the political agenda in Germany. In 2009 the program “Verbundweiterbildung*^plus^* Baden-Württemberg” (VWB*^plus^* BW) was developed by the Competence Center for General Practice in Baden-Wuerttemberg to ensure primary healthcare in the future by enhancing the attractiveness of general medicine. This paper describes the experiences that have been gathered in developing a post-graduate training-program for physicians undergoing specialist training in general practice.

**Project description: **The Competence Center for General Practice in Baden-Wuerttemberg supports the organization of regional networks dedicated to post-graduate medical education. First core element of the VWB*^plus^* BW program is a special seminar series for physicians pursuing post-graduate training. This seminar program is aligned with the German competency-based curriculum in general medicine and is meant to promote medical expertise and other related competencies, such as business and medical practice management and communication skills. Mentoring and advising the physicians regarding professional and personal planning form the second core element. The third core element is seen in the train-the-trainer seminars that address the competencies of the trainers. In order to focus the program’s content closely on the needs of the target groups, scientifically based evaluations and research are carried out.

**Results: **Since starting in 2009, 685 physicians have entered the program and 141 have passed the examination to become medical specialists (as of December 2016). In total, 31 networks, 60 hospitals and 211 general practices have participated. The seminar sessions have been rated on average with 1.43 on a six-point Likert scale by the physician trainees (1=extremely satisfied, 6=extremely dissatisfied). Alongside the medical training, these physicians viewed the exchange of information and experiences with other physicians as very positive and important. In 185 seminars lasting 90 minutes each, the seminar program has presently covered 250 out of 320 units in the competency-based curriculum for general medicine. A total of 281 trainers have been trained in 13 train-the-trainer courses and have rated this course on average with 1.36 on a six-point Likert scale. Above all, the trainers emphasized the exchange of information and experiences with other trainers as very positive. In 2013 the DEGAM concept for its Verbundweiterbildung^plus^ program was developed based on that of the VWB*^plus^* BW. Since 2008 over 40 articles on the topic of post-graduate medical education have been published.

**Conclusion:** The steadily increasing number of participants over the years demonstrates that the VWB*^plus^* BW is relevant for recent medical graduates and contributes to the attractiveness of general practice. The consistently excellent evaluations of the training program and the train-the-trainer course affirm the focus on the needs of the target groups. The post-graduate VWB*^plus^* BW program advances structured, competency-based and quality-oriented specialist training and fosters professional sharing between physicians – something that could also be relevant for other fields. The increasing numbers of participating physicians and specialists in general practice in Baden-Württemberg lead to the conclusion that the VWB*^plus^* BW program positively influences the number of general practitioners.

## Zusammenfassung

**Hintergrund: **Aufgrund des drohenden „Hausarztmangels“ rückt die Versorgung mit Hausärzten und im Zuge dessen die Aus- und Weiterbildung zum Facharzt für Allgemeinmedizin zunehmend auf die politische Agenda in Deutschland. Mit dem Ziel die hausärztliche Versorgung durch eine Steigerung der Attraktivität der Allgemeinmedizin zukünftig sicherzustellen, wurde 2009 das Programm Verbundweiterbildung*^plus^* Baden-Württemberg (VWB*^plus^* BW) im bestehenden KompetenzZentrum Allgemeinmedizin Baden-Württemberg entwickelt. Diese Arbeit beschreibt die Erfahrungen bei der Entwicklung einer curricularen Verbundweiterbildung für Ärzte in Weiterbildung (ÄiW) zum Facharzt für Allgemeinmedizin.

**Projektbeschreibung: **Das KompetenzZentrum Allgemeinmedizin Baden-Württemberg unterstützt den Aufbau und die Ausgestaltung regionaler Weiterbildungsverbünde. Erstes Hauptelement der VWB^plus^ BW ist das begleitende Seminarprogramm für ÄiW. Es ist an den Inhalten des Kompetenzbasierten Curriculums Allgemeinmedizin ausgerichtet und soll medizinische Expertise und weitere ärztliche Kompetenzen wie Kenntnisse in Betriebswirtschaft, Praxisführung und Kommunikation fördern. Zweites Hauptelement stellen individuelles Mentoring und Beratung der ÄiW bei der Berufs- und Lebensplanung dar. Drittes Hauptelement sind Train-the-Trainer-Seminare, bei denen die Kompetenzen der Weiterbilder geschult werden. Um Inhalte eng an den Bedürfnissen der Zielgruppen auszurichten, werden wissenschaftliche Evaluationen und Begleitforschung durchgeführt.

**Ergebnisse: **Seit Beginn im Jahr 2009 sind 685 ÄiW in das Programm eingetreten, von denen 141 ihre Facharztprüfung bestanden haben (Stand Dezember 2016), insgesamt nehmen 31 Verbünde, 60 Krankenhäuser und 211 Hausarztpraxen teil. Die Seminartage werden von ÄiW durchschnittlich mit 1,43 auf einer sechsstufigen Likert-Skala (1=sehr zufrieden bis 6=sehr unzufrieden) beurteilt. Die ÄiW erachteten neben der fachlichen Weiterbildung den kollegialen Austausch als sehr positiv und wichtig. Bisher wurden im begleitenden Seminarprogramm in 185 Seminaren à 90 Minuten 250 von 320 Inhalten des Kompetenzbasierten Curriculums Allgemeinmedizin berücksichtigt. In 13 Train-the-Trainer-Kursen konnten 281 Weiterbilder geschult werden. Sie beurteilten diesen Kurs im Mittel mit 1,36 auf der sechsstufigen Likert-Skala. Die Weiterbilder hoben vor allem den kollegialen Austausch als sehr positiv hervor. 2013 wurde das DEGAM-Konzept Verbundweiterbildung*^plus^* in Anlehnung an die VWBplus BW entwickelt. Seit 2008 wurden über 40 Publikationen zum Thema ärztliche Weiterbildung publiziert.

**Schlussfolgerung: **Die seit Jahren wachsenden Teilnehmerzahlen sprechen dafür, dass die VWB*^plus^* BW für den ärztlichen Nachwuchs relevant ist und zur Attraktivität des Fachs Allgemeinmedizin beiträgt. Dabei belegen die durchgehend sehr guten Evaluationsergebnisse des Schulungsprogramms und des Train-the-Trainer-Kurses die Ausrichtung der Inhalte an die Bedürfnisnisse der Zielgruppen. Durch das curriculare Weiterbildungsprogramm VWB*^plus^* BW werden eine strukturierte, kompetenzorientierte und an Qualität ausgerichtete Weiterbildung sowie der kollegiale Austausch gefördert, was auch für andere Fachrichtungen relevant sein könnte. Die steigende Zahl von ÄiW und Fachärzten für Allgemeinmedizin in Baden-Württemberg lässt darauf schließen, dass die VWB*^plus^* BW die Anzahl der Hausärzte positiv beeinflusst.

## Introduction

In face of the looming **shortage of general practitioners** in Germany primary care provided by general practitioners and, as a result, medical education and post-licensure specialization in general practice have increasingly become a part of the political agenda. While searching for the root causes of this shortage of general practitioners, structural weaknesses in post-graduate education, such as the absence of a curriculum for physicians undergoing post-graduate training, were found and criticized. The unattractiveness of general practice in comparison to other medical specialties was also identified as a reason [1], [2]. Analysis of the German Medical Association (*Bundesärztekammer*) in 2015 shows that for the specialty medical exams taken (*Facharztprüfung*) there has been for years now a steady imbalance between medical specialists and general practitioners of 89.1% (n=10894) to 10.9% (n=1337) [3]. Despite the high number of medical specialists, there has been talk for some time now regarding a general shortage of doctors [4], and there have been discussions of increasing the quality of post-graduate medical education through structured specialized training in all subjects [5].

The **quality of post-graduate medical education** in general practice is under particular scrutiny: the German College of General Practitioners and Family Physicians (DEGAM) commissioned a report in 2008. International experts in medical post-graduate study asserted in 2009 that specialty training programs in general practice in Germany meet none of the European criteria for best practice [6]. In particular, the overall program structure was identified as insufficient and impaired access to rotations of posts to train for general practice was criticized. Furthermore, the lack of focus on competencies and inadequate presentation of theory, for example in seminars, were identified as faults. Other points of criticism included insufficiently available advisors, trainers who had not been trained according to professional teaching standards, and the specialty training’s general lack of relevancy to the future practice of primary care providers [6]. In a survey of physicians undergoing specialty training in general practice, nearly 90% of these physician trainees felt that a structured training program with rotating posts and the acquisition of business management skills as well as interprofessional collaboration in local healthcare networks were (very) important [7]. In another qualitative study, physician trainees identified several barriers, specifically the lack of structure in the different phases of post-licensure training, the low salaries during the out-patient phase of specialty training, and the general conditions, viewed as deterrent due to financial insecurity, that are associated with practicing general medicine in Germany [8].

The program **Verbundweiterbildung*^plus^* Baden-Württemberg** (VWB*^plus^* BW) was created as a measure to counteract the shortage of general practitioners and stands for high-caliber, internationally competitive specialty training in general practice. The aim of this project report is to report on the experiences gathered during the last eight years spent developing a curriculum for a networked training program and to describe new paths in post-graduate medical training in general practice.

## Project description

### Background

From 2005 to 2007, a forum for post-graduate education existed at the Department of General Practice and Health Services Research of the Heidelberg University Hospital. Its goal was to support physicians undergoing specialty training and young medical specialists in general practice by offering seminars in parallel to professional practice, for instance on communication skills [9]. In 2007 the first competence center for general practice in Germany was established at the Heidelberg University Hospital networking the five medical schools in Baden-Württemberg with coordination by the Department of General Practice and Health Services Research [10]. The aim of the **Competence Center for General Practice in Baden-Wuerttemberg **(BW Competence Center) is to ensure future primary healthcare by increasing the attractiveness of general practice. To attain this goal, various projects and measures in the areas of research, education and post-graduate education have been developed and implemented [11]. The pilot project Verbundweiterbildung*^plus^*, developed for the five-year phase of post-graduate education [12], is now known as **Verbundweiterbildung*^plus^* Baden-Württemberg** (VWB*^plus^* BW) and serves as a model for all of Germany.

### The concept behind Verbundweiterbildung^plus^ Baden-Württemberg

VWB^plus^ BW is a curriculum-based post-graduate program for physicians seeking post-licensure qualification in Baden-Württemberg as specialists in general practice. These physicians register voluntarily for the program. As participants they profit from key aspects of the VWB^plus^ BW program (see below) in addition to the medical specialty training received at a hospital, medical practice or other educational institution.

The legal basis for the VWB*^plus^* BW program is formed by the cooperative agreements between the BW Competence Center (representing the Heidelberg University Hospital) and the hospitals or medical practices as employers of the physician trainees. These agreements contain legal provisions regarding program financing, compensation, the number of days allotted for attending the special seminars, and the duties of the BW Competence Center [12]. The BW Competence Center also promotes the establishment and organization of regional post-graduate training networks. Within a given network the objective is to give physician trainees the opportunity to participate in** structured rotations**. To enable this, at least one hospital and multiple medical practices (general practitioners and specialists) join together to provide physician trainees insights into different medical fields and to ensure ongoing training without interruptions when positions are rotated. The **university-based affiliation** of the training networks with the BW Competence Center is meant to guarantee the implementation of current scientific knowledge in routine work and ensure high-caliber training and instruction in the program for specialization in general practice. In addition, this affiliation serves as an “umbilical cord” for physician trainees who are particularly interested in academic work.

The BW Competence Center was initially funded by the Baden-Württemberg Ministry for Science, Research and the Arts. Funding to expand the program in the rural areas was made available by the Baden-Württemberg Ministry for Rural Affairs and Consumer Protection. The Baden-Württemberg Ministry for Social Affairs and Integration gave support for the development and integration of digital teaching and learning programs. In addition, the program also receives support from the AOK Baden-Württemberg, a regional statutory health insurance provider, in the form of extra payment for the P1 billing rates for primary care given by general practitioners and from the Baden-Württemberg Association of General Practitioners (*Hausärzteverband Baden-Württemberg*) for ongoing training for the instructors. In 2015 after public funding ran out, the VWB*^plus^* BW was forced to fund itself. Since then, it has been funded by contributions from participating hospitals (1,500€/year per physician trainee) and medical practices (500€/year per physician trainee). In exceptional cases, the physician trainees cover the costs themselves (500€/year per physician trainee). A total of 31 training networks remain active out of the previous number of 43 prior to the transition in funding. The advantage for the participating practices and hospitals lies especially in the ability to employ well-educated physicians as they undergo their specialist training.

**Figure 1:**
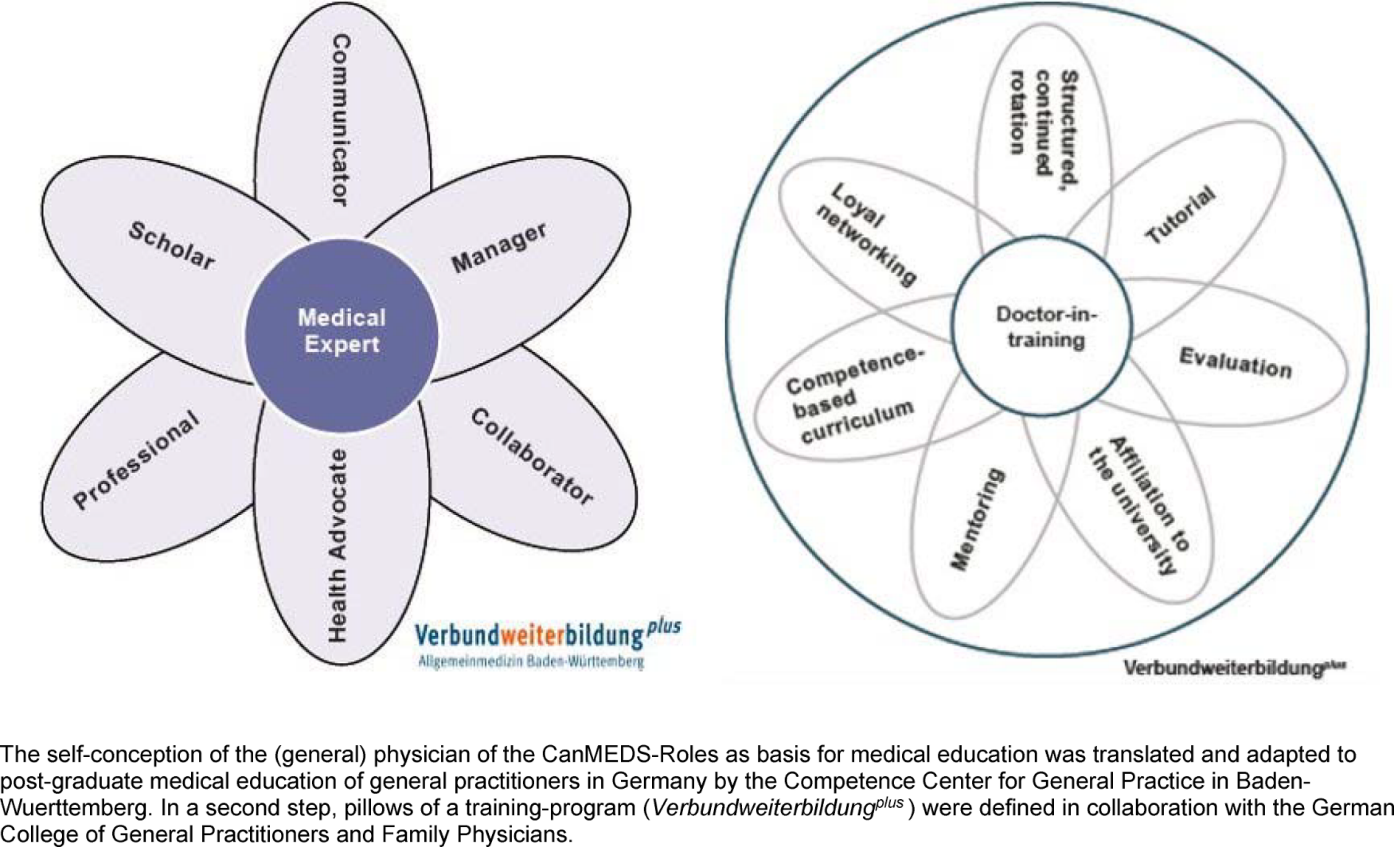
The self-conception of the (general) physician

To ensure the quality of the post-graduate program, the VWB*^plus^* BW draws on a role model of the (general) medical practitioner which was used to adapt the CanMEDS roles for the post-graduate program [13]. This role model goes beyond medical expertise to include skills involving communication, management, collaboration, how to best represent patients, and professionalism (see figure 1 (Fig. 1)).

### The elements of Verbundweiterbildung^plus^ Baden-Württemberg

The VWB*^plus^* BW program consists of three core elements: a seminar program for physicians undergoing specialist training, mentoring and advising for those physician trainees, and train-the-trainer seminars for instructors. In addition, the VWB*^plus^* BW also hosts Landtage (days on which specific rural areas are showcased to promote them as places to practice general medicine) and network gatherings where trainers from hospitals and medical practices can share and exchange information. VWB*^plus^* BW also carries out concomitant research on post-graduate education, including evaluations of the post-graduate program.

One of the three main elements of the VWB*^plus^* BW is the **accompanying seminar program** in which medical expertise (dermatology, otolaryngology, pediatrics, etc.) and general skills e.g. in business management, practice management and communication can be acquired. The seminar program takes place in parallel to the specialty training and is constantly evaluated in terms of content and teaching so that these can be adapted to meet the needs of the attendees [6], [14]. On each seminar day a questionnaire is administered (six-point Likert scale; 1=extremely satisfied, 6=extremely dissatisfied) to evaluate seminar content, presentation, opportunities for participation, atmosphere, and practical relevancy. The questionnaires also have space for open-ended comments for individual comments or topic suggestions. Each year, four day-long seminars are held in Heidelberg consisting of 12 sessions (90 minutes each) and four day-long seminars in Freiburg, Stuttgart, Tübingen and in the region of southern Württemberg consisting of nine seminars (also 90 minutes each). In addition to these individual day-long seminars, the VWB*^plus^* BW offers participating physicians the opportunity once a year to attend a two-day training session (two seminar days with an overnight stay for 24 attendees). These cover a total of six ninety-minute sessions. These seminar sessions are held in a central location in Baden-Württemberg and focus on a particular topic (communication, palliative medicine, or long-term care, etc.) The seminars are set up to be as interactive and relevant to practice as possible, providing attendees a chance to hone their skills through practical exercises in (small) groups. One general goal during these seminars is to foster professional networking among the physician trainees and appropriate amounts of time are planned for this, including general breaks, common meal times, and evening activities for the two-day training sessions.

The topics for the day-long seminars are based on the competency-based curriculum in general medicine. This competency-based curriculum was designed and developed in a collaborative process between the DEGAM, JADE (a network of trainees and young GPs in Germany) and the BW Competence Center. It covers – as a supplement to the logbooks required by the medical associations – the basic skills and competencies defined for general practitioners [15]. These are listed in detail, together with common general medical procedures, and assist those undergoing specialty training and their trainers by enabling the trainee to compare his or her self-assessment with that of the trainers. The competency-based curriculum in general practice is intended to serve as a common thread running through the entire course of the specialty training to help physicians focus on the information relevant to general practice [15], [16].

With the aim of encouraging physicians pursuing post-licensure training to set up practice in rural areas, ***Landtage*** are held once or twice a year. During these locally-held events representatives of rural regions – doctors, mayors, district administrators, etc. – have the opportunity to come into contact with physicians still undergoing specialty training. The main objectives are to communicate expectations, dispel preconceptions on both sides, and become familiar with the life of a country doctor (the wide range of care, new healthcare concepts, etc.) [17].

The second core element of the VWB*^plus^* BW program is the **mentoring** and advising. The employees of the VWB*^plus^* BW function as general contact partners for all those involved and are available to advise the physician trainees during the entire post-graduate program. In addition, the BW Competence Center offers individualized counseling with the goal of supporting the physician trainees as they make decisions about their future professional and personal lives. Members of the VWB*^plus^* BW who are experienced specialists in general practice are available via email, telephone or for in-person meetings [18]. A main focus is placed on advising those who are re-entering the medical profession after a longer hiatus and those coming into the profession via a less traditional path, meaning specialists in other areas of medicine who wish to add a specialty in general practice [19]. Since the end of 2016 “group mentoring” has been offered during the two-day seminars during which a group of physician trainees shares information under the moderation of an experienced mentor and psychotherapeutic supervision.

Since 2011 the third core element of the VWB*^plus^* BW program has been the** train-the-trainer courses** as a measure to prepare the instructors. During these two-day sessions with an overnight stay, aspects of the post-graduate program pertaining to teaching, organization and the relevant rules and regulations are covered in detail [20], [21]. The content is aligned with the needs of the trainers, who are surveyed in advance [22], [23]. A special feature is evident in the use of actors to simulate patients as a way to practice giving professional feedback [21]. Other pertinent topics are also addressed, such as that of finding a physician to take over an existing medical practice.

The **network gatherings** are intended to encourage cross-sector exchange between trainers from hospitals and trainers from medical practices. To ensure structural integrity and quality assurance, once each year all of the people involved in the post-graduate training networks, trainers from hospitals and practices, and the physician trainees are invited to come and hear about new developments regarding VWB*^plus^* BW, and meet with each other to discuss these changes and other topics, such as the role of the trainers in respect to Gen X and the Millennials or the practical implementation of good teaching and training practices as part of daily medical routines.

Further strategies of the VWB*^plus^* BW to promote quality assurance are embodied in the** regular evaluations of and scientific research** on all the program’s components. These activities make it possible to constantly adjust the program’s focus to match the desires and needs of the participants, with regard for the competency-based general practice curriculum and the latest scientific knowledge. To accomplish this, regularly scheduled qualitative and quantitative surveys are conducted among the physician trainees and the trainers depending on the research focus.

## Results

### The concept behind Verbundweiterbildung^plus^ Baden-Württemberg

**Figure 2:**
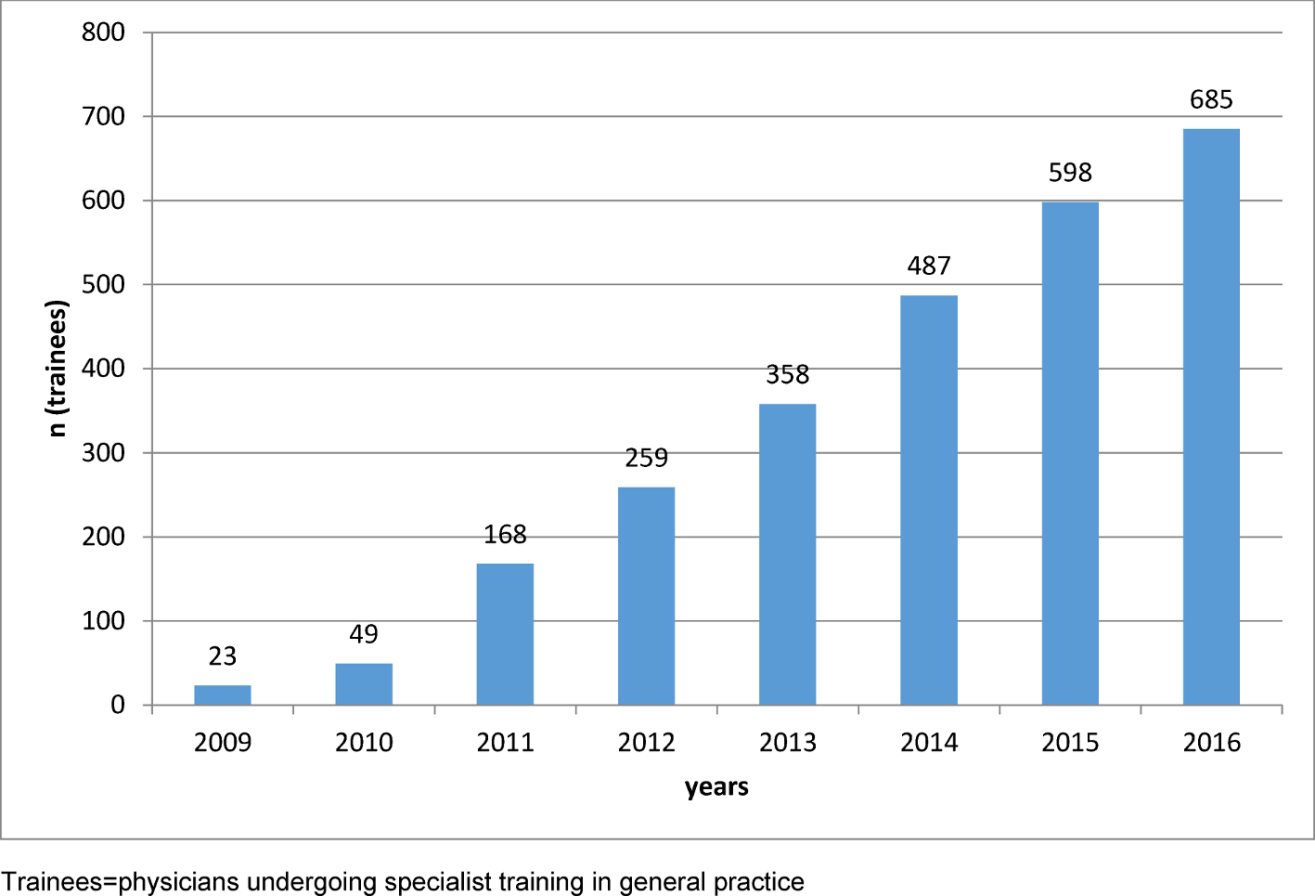
Total number of post-graduate trainees in the Verbundweiterbildung^plus^ Baden-Württemberg since its start in 2009.

Since its begin in 2009, a total of 685 physician trainees have entered the VWB*^plus^* BW program; the growth in participants over time is shown in figure 2 (Fig. 2). Table 1 (Tab. 1) gives an overview of the numbers for VWB*^plus^* BW through the end of December 2016. At the end of 2016, 141 physician trainees had successfully passed the exam to become medical specialists for General Practice (*Facharzt*). The number of network members with active cooperative agreements is 31, the number of hospitals 60 and medical practices 211. The cooperative agreements have provisions for increasing the physician trainee salaries during the out-patient training phase so that they match those of the municipal wage agreements for physicians. The agreements also include a contractual commitment to allot 10 days per year instead of three for parallel training to enable regular attendance of the VWB*^plus^* BW’s special seminars and other professional conferences and congresses.

In 2010 the VWB*^plus^* BW received the award for being an outstanding location (*Ausgezeichneter Ort 2010*) by “Germany, the Land of Ideas” – an initiative to promote and encourage the realization of outstanding projects in Germany and around the world. After a four-year set-up phase, the VWB*^plus^* BW received formal expert attestation in 2012 confirming that it is the only post-graduate study program in general practice in Germany that meets the European best-practice criteria [24]. In 2013 the DEGAM concept for networked post-graduate training (Verbundweiterbildung*^plus^*) was developed based on the program of the Competence Center for General Practice in Baden-Wuerttemberg [25].

### The elements of Verbundweiterbildung^plus^ Baden-Württemberg

In the **accompanying seminar program** since 2009 a total of 185 difference seminar topics (see table 1 (Tab. 1), Attachment 1 and Attachment 2) have been presented in 90-minute sessions on 25 seminar days per year. As a result, it has been possible to cover **250 of 320 curricular units of the competency-based general practice curriculum**, plus additional topics such as telemedicine and healthcare for asylum seekers. At Attachment 1 and Attachment 2 and also table 2 (Tab. 2) and table 3 (Tab. 3) list the competency-based curricular topics covered in the special seminars (see Attachment 1 and Attachment 2, see table 2 (Tab. 2) and table 3 (Tab. 3)). A good 10% of the general practice seminar topics (48 of 418) were presented by physician trainees themselves in the form of peer-to-peer instruction. This number is increasing overall; in 2016, eight of 26 seminar presenters were physician trainee participants in the VWB*^plus^* BW program.

On eight training days each year up to 121 physician trainees attend sessions in Heidelberg and up to 81 at other sites; up to 30 physician trainees per year attend the two-day seminars. Per year each physician trainee is allowed to participate in a total of four day-long seminars and one two-day seminar. When registering for these seminars, the physician trainees tend to prefer medical topics such as dermatology or pediatrics, despite expressing a desire for organizational topics such as medical billing or the principles of business management.

The evaluation of the VWB*^plus^* BW program has been described by Flum et al. [14]. The **special seminar series** have been rated on average from the beginning very positively with 1.43 based on a six-point Likert scale (1=extremely satisfied, 6=extremely dissatisfied). In response the organization and the seminar content have been steadily adapted to meet the needs of the physician trainees. The overall impression of each seminar session has been evaluated by the physician trainees as being between 1.29 and 1.57 for the entire period. The organization and materials were rated with 1.3 to 1.5 and 1.53 to 1.82, respectively. The professional exchange during the seminars was rated on average over the years as being between 1.17 and 1.46, whereby the two-day seminars were rated the highest by VWB*^plus^* BW participants [14]. The physician trainees viewed both the professional exchange and the post-licensure medical training very positively and considered them important. Feedback from the physician trainees on the day-long seminars specifically praises the** balance between post-licensure medical training and professional interaction**. The enthusiasm for the field of general practice and practicing as a GP was identified as standing out positively; the day-long seminars were experienced as motivation to push forward with their own professional development.

The mentoring offered by the VWB*^plus^* BW program has been described by Hoffmann et al. [18] who have been able to show that advising and **mentoring** the physician trainees was received positively and, above all, taken advantage of in regard to questions involving labor law, organizational issues and personal matters [18]. The VWB*^plus^* BW program has also been able to reach out to physicians seeking a specialty in general practice via a less common path [19].

The train-the-trainer seminars have been described by Ledig et al. [21]. Since its start, 13 two-day sessions have been held, and a total of 281 physicians have participated in the VWB*^plus^* BW’s **train-the-trainer course** and rated it very positively with an average of 1.4 on a six-point Likert scale (1=extremely satisfied, 6=extremely dissatisfied) [21]. The different aspects were evaluated as follows: informative content 1.5 to 1.6, presentation 1.4 to 1.6, opportunity to participate 1.2 to 1.5, atmosphere 1.2 to 1.4, and practical relevance 1.4 to 1.5. In the open-ended responses, the evaluations primarily reflected praise for the use of standardized patients (actors) for giving feedback. This program has enabled the Competence Center for General Practice in Baden-Wuerttemberg to better recruit and retain experienced trainers. This aspect and the opportunity for professional exchange were evaluated as being very beneficial and motivational by the participants.

The annual **network gatherings** were received positively as a forum for finding out about changes and developments in the program.

Regular **evaluations and scientific research** have been successfully established: all components of the VWB*^plus^* BW program are routinely evaluated using surveys or interview studies. Since 2008 over 40 publications on topics in post-graduate education have been written at the Competence Center for General Practice in Baden-Wuerttemberg and its **public relations** campaign is an active one. At the national level the VWB*^plus^* BW is regarded as a “milestone project.” The knowledge derived from research and study is regularly presented at national and international conferences and congresses.

After completing the VWB*^plus^* BW program, graduates who have passed the medical specialist exam (*Facharztprüfung*) still wish to participate in the special seminars. To satisfy this need,** alumni reunions** have been held since 2016 serving as opportunities for professional networking among graduates and continuing education – particularly on issues surrounding establishing and organizing a medical practice.

To further integrate this post-graduate program into an even larger network, fifth-year medical students who are interested in pursuing a post-licensure specialty in general practice are increasingly attending the special VWB*^plus^* BW seminars.

## Discussion

With the VWB*^plus^* BW it has been possible to establish a steadily growing, competency-based post-graduate program for general practitioners-to-be at the BW Competence Center. Part of the basis for this is the successful creation of networks and cooperation with hospitals and medical practices. After public funding was no longer available, the continued existence of the VWB*^plus^* BW program, a recognized pilot project, has been made possible through the commitment of the participating hospitals and medical practices. The number of VWB*^plus^* BW program participants has grown for years, and the feedback given by the physician trainees and trainers is continually very positive. The VWB*^plus^* BW’s special seminars have covered the majority of topics included in the competency-based general practice curriculum, plus additional content. In addition, the basic elements of the VWB*^plus^* BW have been successfully anchored in the form of professional networking, mentoring and advising, train-the-trainer courses, ongoing evaluations and quality assurance.

Across Germany the VWB*^plus^* BW program exerts influence on the development of other programs and post-graduate training networks. As an example, in 2013 the concept of DEGAM’s Verbundweiterbildung*^plus^* program took on the basic program elements without making any changes [25]. In the meantime, other competence centers for general practice, similar to the BW Competence Center, have been established in Germany, for instance in North Rhine-Westphalia in 2012, Hessen in 2013, Mecklenburg-Western Pomerania in 2016 and Thuringia in 2016. Our experiences could assist not only other general practice competency centers presently being established, but also post-graduate study programs in other subject areas.

In Baden-Württemberg between 2011 and 2015, approximately 680 physician trainees have successfully sat for and passed the exam for specialization in general practice, with the number of administered exams increasing from 108 in 2011 to 192 in 2015 [https://www.aerztekammer-bw.de/40presse/05aerztestatistik/05a.pdf (letzter Zugriff 20.10.2016)]. In addition, the number of subsidized training positions in general practice and the number of subsidized physician trainees (some part-time) have doubled with the Baden-Württemberg Association of Statutory Health Insurance Physicians (*Kassenärztlichen Vereinigung Baden-Württemberg*) between 2010 (184 subsidized positions=full-time equivalents and 352 physician trainees) and 2015 (383 positions=full-time equivalents and 719 physician trainees) [http://www.kbv.de/html/ (letzter Zugriff 07.04.2017)]. A study shows that many of the physician trainees in the VWB*^plus^* BW program remain in Baden-Württemberg [26][. With a constantly increasing number of participants in the VWB*^plus^* BW program and with now over 141 specialist physicians, it can be assumed that the VWB*^plus^* BW program clearly contributes to this positive effect.

In order to continue this upward trend in post-graduate medical education, it will be necessary to find permanent funding for university-based post-licensure programs like the VWB*^plus^* BW. These programs must be based on up-to-date scientific knowledge. Requirements for such programs have been jointly articulated with DEGAM [27]. In addition, a working group associated with DEGAM’s continuing education committee is presently drafting quality indicators for future accreditation of post-licensure training networks in DEGAM’s Verbundweiterbildung*^plus^*; these indicators are based on international standards [28]. We hope that this will further ensure the professionalism and quality of post-graduate medical education in Germany and thus increase the attractiveness of general practice.

### Strengths/limitations

One of this project description’s strengths is that it is an overview of a successful post-graduate medical program. Some of the discoveries reported as results refer to the experiences of physician trainees and VWB*^plus^* BW employees which were not recorded within the scope of a scientific study. The program is located in Baden-Württemberg. Despite high numbers of participants, the results can be applied only to a limited extent to Germany or other countries. The scientific research conducted on VWB*^plus^* BW program is made difficult in that only rarely is data available for comparison with other (German) cohorts.

## Conclusion

The growing numbers of participants over the years show that the concept of the VWB*^plus^* BW program is relevant for new medical graduates and contributes to the attractiveness of general practice. The overall very positive evaluation results for the accompanying seminar series and the train-the-trainer course demonstrate that the content focuses on the needs of the target groups. Post-graduate programs such as the VWB*^plus^* BW promote structured, competency-based and quality-oriented training, something that may also have relevance for other fields. The current developments in Baden-Württemberg regarding subsidized physician trainees and specialists in general practice lead to the conclusion that the VWB*^plus^* BW program has positively influenced the number of general practitioners.

## Acknowledgement

The authors wish to thank all trainers and participants engaged in the VWB*^plus^* BW program.

## Competing interests

All of the authors have worked as part of the VWB*^plus^*. The authors declare that they have no competing interests. 

## Supplementary Material

Curricular units of part I competency-based general practice curriculum

Curricular units of part II competency-based general practice curriculum

## Figures and Tables

**Table 1:**
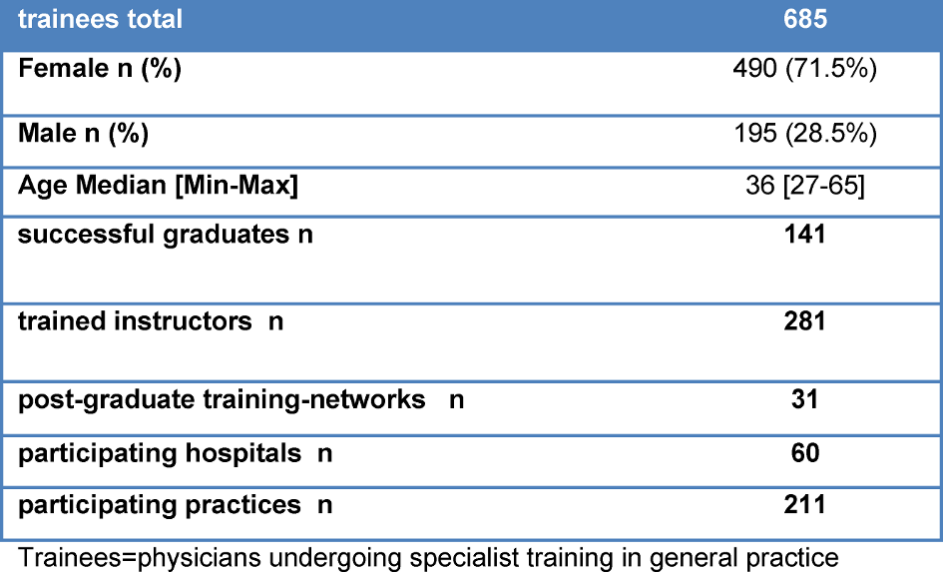
Operating figures of the Verbundweiterbildungs^plus^ Baden-Württemberg (December 31^th^ 2016)

**Table 2:**
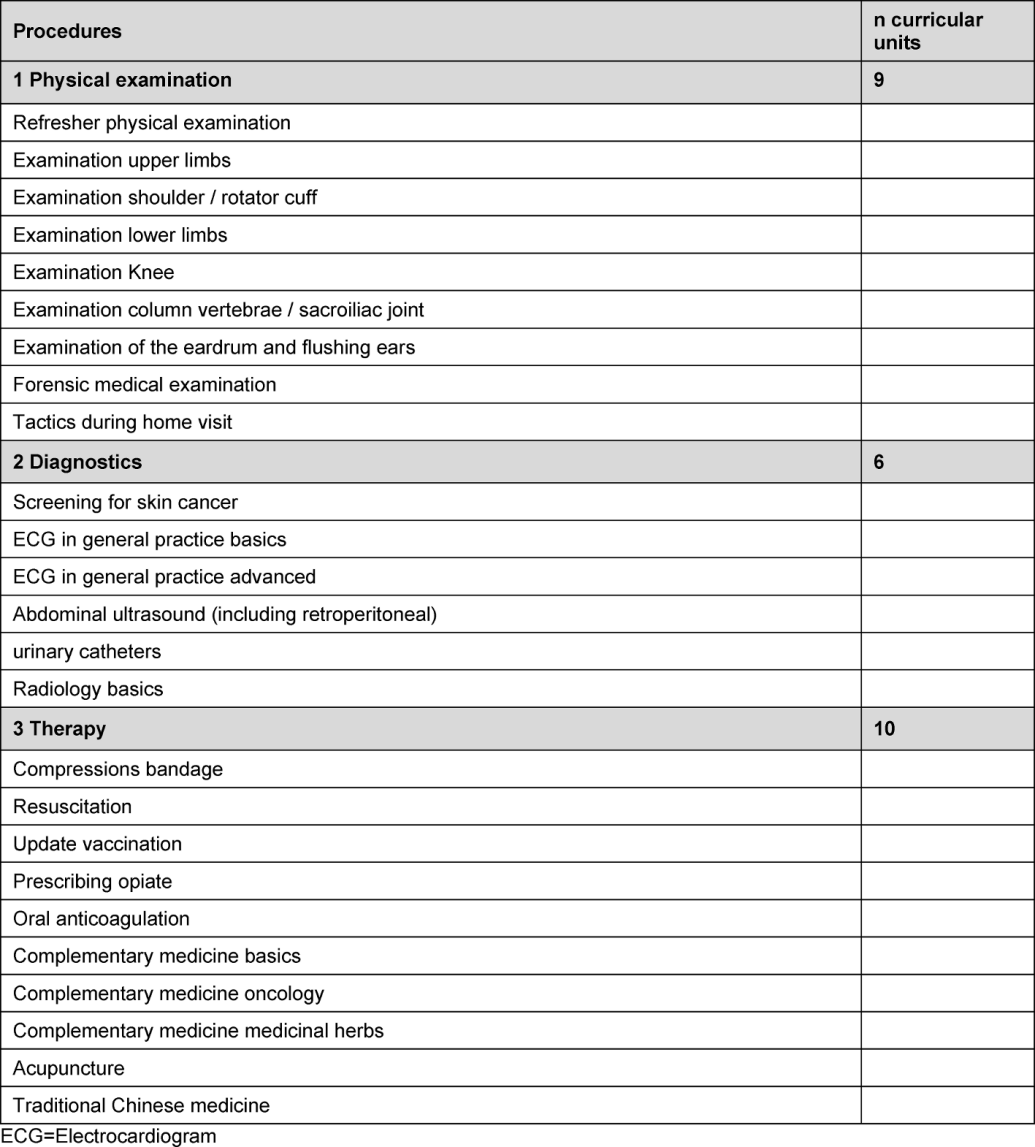
Curricular units of part III competency-based general practice curriculum

**Table 3:**
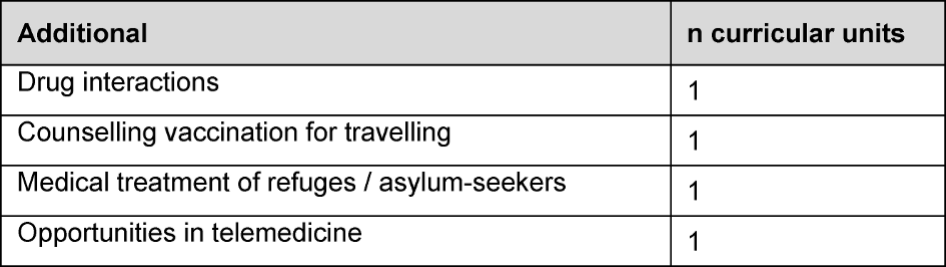
Curricular units which are not part of the competency-based general practice curriculum
