# Supplementary material for: New paths in post-graduate medical training in general practice – 8 years of experience with the pilot project Verbundweiterbildungplus Baden-Württemberg
Source: GMS J Med Educ. 2017 Nov 15;34(5):Doc62. doi: 10.3205/zma001139 (PMC5704616; doi:10.3205/zma001139)
Supplement: Curricular units of part I competency-based general practice curriculum [file JME-34-62-s-001.pdf]

**Medical Expert**	**n curricular units**
**1 Metabolism, Nutrition, Gastrointestinal**	**11**
Diabetes mellitus: Diagnosis	
Diabetes mellitus: Therapy	
Diabetes mellitus: (initiating) Insulin-Therapy	
Diseases of the thyroid gland	
Irritable bowel disorder	
Proctology	
Microbiome	
Travelling diseases	
Diverticulitis	
Cholecystolithiasis/-cystitis	
Endocrinology in general practice	
**2 Respiratory tract and ears**	**5**
Common cold	
Differential diagnosis: Cough	
Advising patients with asthma / COPD	
Sleep apnea syndrome	
The aching ear	
**3 Cardiovascular system**	**4**
Arterial Hypertonia	
Venous diseases	
Thoracic pain in the general practice	
Cardiology in general practice	
**4 Musculoskeletal system, pain, injuries**	**10**
Neck pain	
Back pain	
Orthopedics: Shoulder	
Orthopedics: Elbows and hands	
Orthopedics: Vertebral column and sacroiliac pain	
Orthopedics: Pool and hips	
Orthopedics: Knee	
Manual medicine / diagnostic and therapy	
Osteoporosis	
Rheumatic diseases	

**5 Eyes and nervous system**	**3**
The red eye / foreign body	
Dizziness	
Head aches	
**6 Sexuality / family planning / reproductive organs / urinary tract**	**9**
Gynecology for general practice	
Oral contraception / morning-after pill	
Supporting pregnancies	
Medication during pregnancy	
Obstetrics for general practice	
Breast-feeding	
Treating the whole family	
Urology for general practice	
Incontinence	
**7 skin**	**8**
Chronic wounds: part 1	
Chronic wounds: part 2	
Overview dermatology	
Acute diseases of the skin	
Common dermatosis in the face and on the head	
Common dermatosis in infants, children and youth	
Common dermatosis in summertime	
Allergology	
**8 Changing awareness, thinking and sensation / psychosocial aspects**	**14**
Psychiatric diseases in general practice	
Psycho-trauma	
Depression	
Diagnostic of depression	
Anxiety disorder	
Guideline-based management of anxiety disorders in general practice	
Somatoform disorders	
Biopsychosocial model in medicine	
Diagnostic, therapy and treating alcohol abuse	
Addiction medicine	
Integration of substitution-therapy	

Dementia	
Guidelines: dementia	
Guidelines: Fatigue	
**9 Pediatrics**	**5**
The feverish child.	
Pediatric emergencies	
Domestic violence / violence against children	
ADHD	
Common check-ups for children (*U-Untersuchungen*)	
**10 Support the chronic ill / multimorbidity**	**9**
Geriatrics	
Transition from practice to nursing home	
Caring for patients in nursing homes	
Agitated patients in nursing homes	
Geriatric Assessment	
Geriatrics in Baden-Württemberg	
Polypharmacy	
Pharmaceutical interactions	
Brown bag review	
**11 palliative medicine**	**5**
Control of symptoms and pain	
Palliative medicine	
Hospices	
Support dying patients / communication with relatives	
Prophylaxis of burn-out in palliative medicine	
**12 Emergencies**	**4**
Emergencies in general practice	
Ready for out-of-hour-care: part 1	
Ready for out-of-hour-care: part 2	
Ready for out-of-hour-care: part 3	

ADHD=Attention deficit hyperactivity disorder, COPD=chronic obstructive pulmonary disease
