# Supplementary material for: New paths in post-graduate medical training in general practice – 8 years of experience with the pilot project Verbundweiterbildungplus Baden-Württemberg
Source: GMS J Med Educ. 2017 Nov 15;34(5):Doc62. doi: 10.3205/zma001139 (PMC5704616; doi:10.3205/zma001139)
Supplement: Curricular units of part II competency-based general practice curriculum [file JME-34-62-s-002.pdf]

Competencies regarding Can-MEDS	n curricular units
**1 Communicator**	**12**
Doctor-Patient communication	
Inter-professional communication	
Inter-cultural communication	
The art of consultation	
The complicated patient	
Motivational communication: alcoholism	
Motivational communication: change of life-style	
Managing conflicts	
Training in de-escalation	
Balint-groups: introduction	
Balint-groups: meeting	
Training in communication: MAAS-Global	
**2 Collaborator**	**7**
Team leadership	
Physiotherapy	
Inter-professional collaboration	
Error handling	
Delegation of duties: Case Management	
Continuity in care: managing admission and discharge from hospital	
Subscription of technical aids	
**3 Manager**	**18**
Managing a practice	
Management: Administration	
Organization of a practice	
Founding a (new) practice	
Business administration for general practice: part 1	
Business administration for general practice: part 2	
Modern general practice: part 1	
Modern general practice: part 2	
Billing in general practice	
Billing of special trainings in general practice	
Ensuring economics	
Marketing your practice	
Insurances for practices	
Qualitative management	
Getting into practice	
Ways of working together: different collaborations	
Taking over a practice	
Financing a practice	

**4 Advocacy of patients**	**14**
Rights of patients (*Patientenrechtegesetz)*	
Patient decree	
Personal caretaker / legal care	
Social medicine: inability to work	
Social medicine: certificates and applications	
Improving care from patients-perspective	
Judging fitness to drive	
Advising donation of organs	
Advising risks: ARRIBA-Score	
Support in patients with cancer (*Krebsinformationsdienst*)	
Health promotion	
Fitness and physical activity	
Social medicine: Part 1	
Social medicine: Part 2	
**5 Learning and Teaching**	**11**
Critical reading	
Evidence based Medicine	
Searching literature	
Diagnostic difficulties in general practice	
Alternatives to representatives of pharmaceutical industries	
Guidelines in general practice	
Journal Club	
Psychology during examinations	
Simulating board-examination	
Use of video-analysis in training	
E-Portfolio	
**6 Professionalism**	**8**
The inner critic	
Legal Do’s and Don’t’s in general practice	
Self-care and managing stress	
Family medicine	
Malpractice	
Working for German medical self-administration / parliaments	
Anatomy of German health-system	
Basics in health economics	

MAAS-Global=Maastricht History-taking and Advice Scoring list consisting of global items
